# Making Accelerating Medicines Partnership Data Findable and Interoperable through a Common Data Model: Extending OMOP for Multi-Source Multimodal Data

**DOI:** 10.64898/2026.08.31.26361831

**Published:** 2026-09-02

**Authors:** Cole Tindall, Rodney Alan Long, Bart Naughton, Brandy M. Mapes, Dave Vismer, Halcyon G. Skinner, Jessica Malenfant, Mano R. Maurya, Mike A. Nalls, Srinivasan Ramachandran, Trang Nguyen, Mette A. Peters, Richard H. Scheuermann

## Abstract

SysBio FAIRplex is a Common Fund Venture Program ^1^ that catalogs and indexes data from the Accelerating Medicines Partnership® (AMP®) Program ^2^ through a federated model in which data hosts retain custody of their datasets. The central piece of this work is the SysBio Common Data Model (SysBio CDM). AMP is a precompetitive public-private partnership started in 2014 that unites the resources of NIH and private partners to improve our understanding of disease pathways and transform current models for developing new treatments by:

- identifying new targets, biomarkers, and development paradigms;
- developing leading-edge tools and technologies;
- collecting large-scale datasets and supporting analytics for open analysis by the public; and
- generating consensus platforms and procedures.

A multidisciplinary Task Force was chartered to design the SysBio CDM by extending the Observational Medical Outcomes Partnership (OMOP) Common Data Model ^3^ into the -omics domain. The Task Force produced a Minimum Viable Product comprising nine OMOP tables; four extension tables for assay and file metadata; and a Common Data Element (CDE) Registry to specify field semantics. This manuscript describes the deliverable: the underlying design choices, the criteria applied in selecting and constructing the extension tables, how the extended model supports multimodal data integration across AMP projects, and what further work to support additional -omics modalities would entail. As an auxiliary methodology, the paper also describes the AI-assisted CDE harmonization workflow used to populate the model.

## Full Text Availability

The license terms selected by the author(s) for this preprint version do not permit archiving in PMC. The full text is available from the preprint server.

